# Effect of changes to Modified Look-Locker Inversion (MOLLI) sequence on T1 Mapping at 3.0 Tesla in healthy volunteers

**DOI:** 10.1186/1532-429X-16-S1-P3

**Published:** 2014-01-16

**Authors:** Adam K McDiarmid, David M Higgins, David P Ripley, Akhlaque Uddin, Ananth Kidambi, Peter P Swoboda, Tarique A Musa, Bara Erhayiem, John P Greenwood, Sven Plein

**Affiliations:** 1 Multidisciplinary Cardiovascular Research Centre & The Division of Cardiovascular and Diabetes Research, Leeds Institute of Genetics, Health & Therapeutics, University of Leeds, Leeds, West Yorkshire, UK; 2 Philips Healthcare, Guildford, Surrey, UK

## Background

Diffuse myocardial fibrosis may be quantified with cardiovascular magnetic resonance (CMR) by calculating extra-cellular volume (ECV) from native and post-contrast T1 values. T1 values are affected by field strength and acquisition sequence. The Modified Look Locker Inversion Recovery (MOLLI) method of T1 mapping is a widely used approach, but many different acquisition schemes have been proposed. Flip angle and the effects of the prior inversions have both been suggested as potential targets for change in MOLLI scheme. In a 3,3,5 MOLLI scheme the third acquisition may be influenced by the two previous magnetisation inversions and incomplete recovery. 3,5 may be used as an alternative and performs similarly to 3,3,5. However a 5,3 scheme offers more points unaffected by prior magnetisation and should maximise signal to noise (SNR) in native myocardium. The equation α _SNRmax=arccos(((T1-T2))/((T1+T2))) allows determination of flip angle for maximal signal in a given tissue for bSSFP readout, which is used by MOLLI. For myocardium this αSImax = 36°, where as blood pool signal is maximal at 50°. As signal is lower in native myocardium an appropriate flip angle should be selected to maximise this, before consideration of off-resonance effects on flip angle selection. Therefore we aimed to assess the effect of changes in MOLLI sequence on the accuracy of T1 mapping in healthy volunteers.

## Methods

23 healthy volunteers were studied on a 3.0 Tesla (Philips Achieva TX) CMR system. Native T1 and 15 minute post-contrast (Gadovist 0.15 mmol/kg) T1 maps were generated from mid-LV short axis with three different MOLLI protocols: 3,3,5 (flip angle 50°); 3,3,5 (flip angle 35°); 5,3 (flip angle 35°). All acquisitions used minimum 3 -second pauses between sets of acquisitions. TR/TE/voxel were 2.2 ms/0.96 ms/1.98 × 1.98 × 10 mm. T1 measurements were made in the inter-ventricular septum. Means and standard deviations were compared between MOLLI T1 estimates and ECV calculated.

## Results

23 volunteers (13 Male:10 Female; mean age 45 ± 11 yrs; mean BSA 1.95 ± 0.21) T1 values and ECV were normally distributed and are as displayed below.

## Conclusions

5,3 MOLLI acquisition resulted in higher native T1 value with lower standard deviation than 3,3,5 scheme with either 35 or 45 flip angle. 5,3 reduces T1 underestimation associated with a traditional 3,3,5 MOLLI scheme. Subsequent calculated ECV has lower standard deviation. 5,3 MOLLI acquisition is shorter than 3,3,5, this may help minimise respiratory artefact in T1 and ECV maps when used in clinical examinations.

## Funding

SP and JPG receive an educational research grant from Philips Healthcare. SP is funded by a British Heart Foundation fellowship

**Table 1:** 

	Native T1 ms (sd)	T1 15 min post contrast ms (sd)	% ECV (sd)
3,3,5 50 flip angle	1113 (± 69)	484 (± 59)	26.0 (± 5.3)

3,3,5 35 flip angle	1158 (± 70)	494 (± 49)	28.0 (± 5.7)

5,3 35 flip angle	1205 (± 36)	503 (± 58)	25.6 (± 3.0)

